# Supplementary material for: Enhancing Water Status and Nutrient Uptake in Drought-Stressed Lettuce Plants (Lactuca sativa L.) via Inoculation with Different Bacillus spp. Isolated from the Atacama Desert
Source: Plants (Basel). 2024 Jan 6;13(2):158. doi: 10.3390/plants13020158 (PMC10818642; doi:10.3390/plants13020158)
Supplement: Supplementary file 1 [file plants-13-00158-s001.zip › plants-2773557-supplementary.pdf]

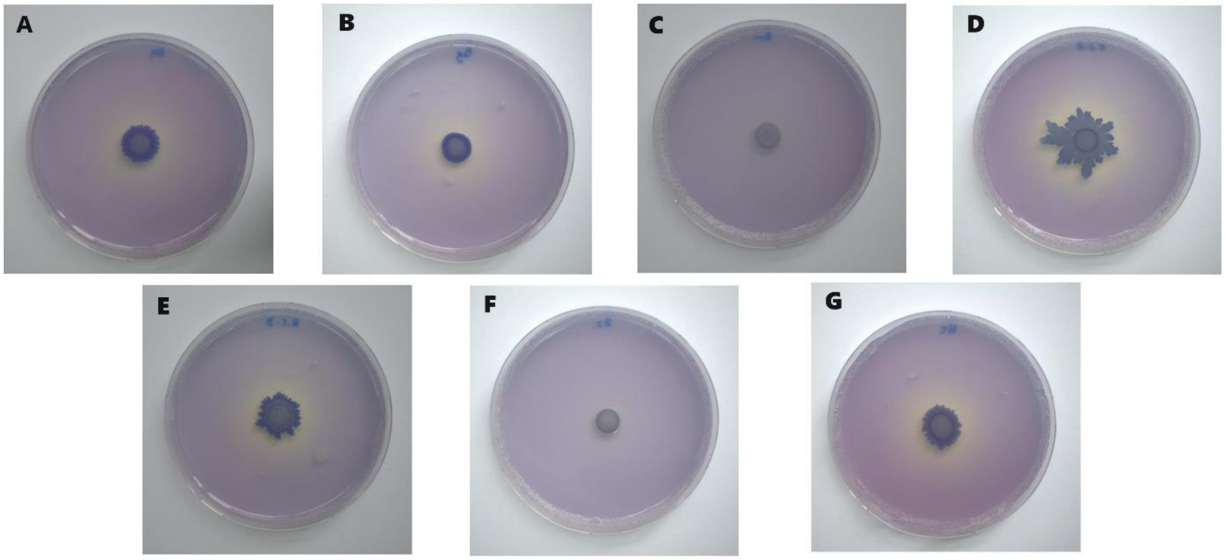

Figure S1. Phosphate solubilization produced by the *Bacillus spp.* strains isolated from the Atacama Desert. (**A**) *Bacillus atrophaeus* ATNJC12015, (**B**) *Bacillus ginsengihumi* ATNJC22015, (**C**) *Bacillus megaterium* ATMLC22021, (**D**) *Bacillus subtilis* ATMLC92021, (**E**) *Bacillus subtilis* ATMLC152021, (**F**) *Bacillus frigoritolerans* ATMLC42021, and (**G**) *Bacillus tequilensis* ATMLC102021.

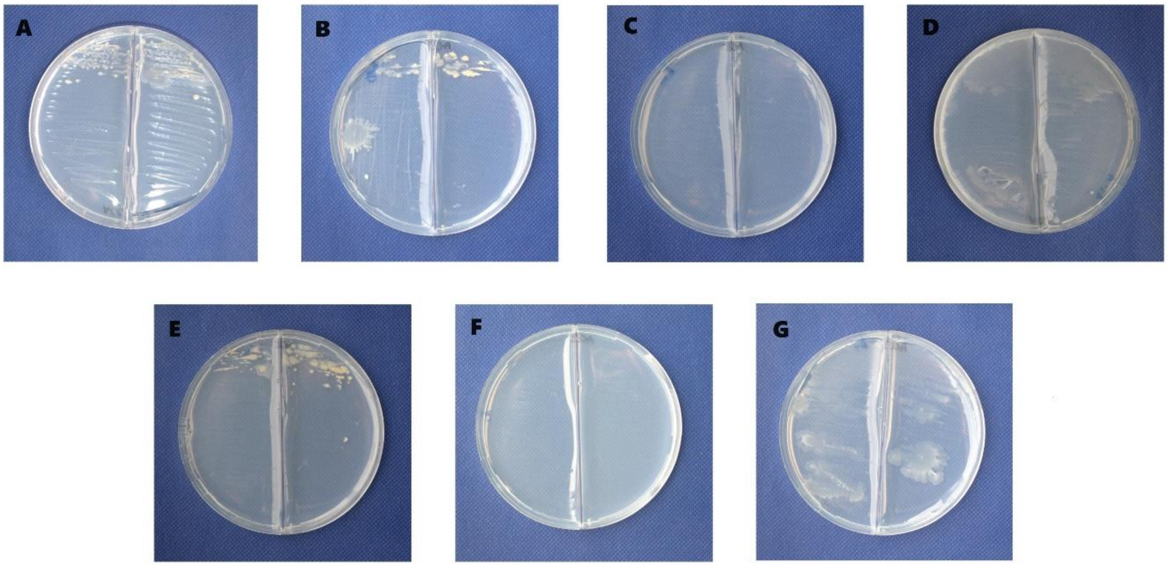

Figure S2. 1-aminocyclopropane-1-carboxylic acid (ACC) deaminase activity produced by the *Bacillus spp.* strains isolated from the Atacama Desert. (**A**) *Bacillus atrophaeus* ATNJC12015, (**B**) *Bacillus ginsengihumi* ATNJC22015, (**C**) *Bacillus megaterium* ATMLC22021, (**D**) *Bacillus subtilis* ATMLC92021, (**E**) *Bacillus subtilis* ATMLC152021, (**F**) *Bacillus frigoritolerans* ATMLC42021, and (**G**) *Bacillus tequilensis* ATMLC102021.

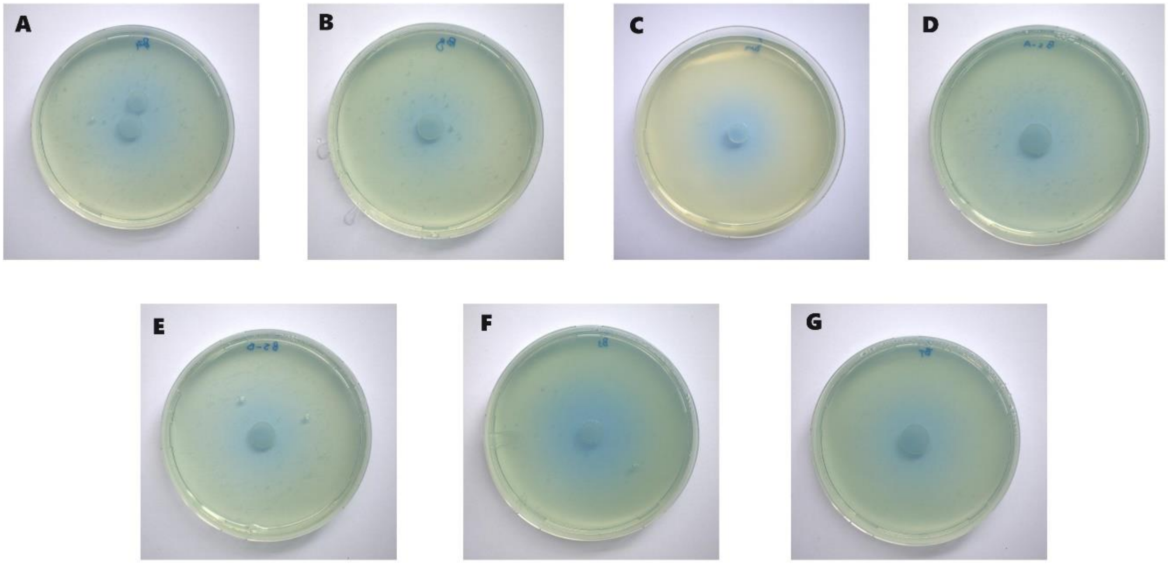

Figure S3. N_2 fixation activity of the *Bacillus spp.* strains isolated from the Atacama Desert. (**A**) *Bacillus atrophaeus* ATNJC12015, (**B**) *Bacillus ginsengihumi* ATNJC22015, (**C**) *Bacillus megaterium* ATMLC22021, (**D**) *Bacillus subtilis* ATMLC92021, (**E**) *Bacillus subtilis* ATMLC152021, (**F**) *Bacillus frigoritolerans* ATMLC42021, and (**G**) *Bacillus tequilensis* ATMLC102021.
